# Deep learning-based segmentation and strain analysis of left heart chambers from long-axis CMR images

**DOI:** 10.1093/ehjimp/qyaf070

**Published:** 2025-06-03

**Authors:** Jonas Leite, Emilie Bollache, Vincent Nguyen, Moussa Gueda Moussa, Thomas Wallet, Mikaël Prigent, Khaoula Bouazizi, Mohamed Zarai, Jérôme Lamy, Perrine Marsac, Niki Procopi, Michel Zeitouni, Alban Redheuil, Antonio Gallo, Elie Mousseaux, Gilles Montalescot, Thomas Dietenbeck, Nadjia Kachenoura

**Affiliations:** Laboratoire D’Imagerie Biomédicale (LIB), Sorbonne Université, INSERM, CNRS, Paris, France; Laboratoire D’Imagerie Biomédicale (LIB), Sorbonne Université, INSERM, CNRS, Paris, France; Laboratoire D’Imagerie Biomédicale (LIB), Sorbonne Université, INSERM, CNRS, Paris, France; Sorbonne Université, Institute of Cardiometabolism and Nutrition (ICAN), Paris, France; Laboratoire D’Imagerie Biomédicale (LIB), Sorbonne Université, INSERM, CNRS, Paris, France; Laboratoire D’Imagerie Biomédicale (LIB), Sorbonne Université, INSERM, CNRS, Paris, France; Sorbonne Université, ACTION group, Pitié-Salpêtrière Hospital (AP-HP), Paris, France; Sorbonne Université, Institute of Cardiometabolism and Nutrition (ICAN), Paris, France; Laboratoire D’Imagerie Biomédicale (LIB), Sorbonne Université, INSERM, CNRS, Paris, France; Sorbonne Université, Institute of Cardiometabolism and Nutrition (ICAN), Paris, France; Sorbonne Université, Institute of Cardiometabolism and Nutrition (ICAN), Paris, France; European Hospital Georges Pompidou, Assistance Publique – Hôpitaux de Paris (AP-HP)/PARCC, Université Paris-Cité, INSERM, Paris, France; Laboratoire D’Imagerie Biomédicale (LIB), Sorbonne Université, INSERM, CNRS, Paris, France; Sorbonne Université, ACTION group, Pitié-Salpêtrière Hospital (AP-HP), Paris, France; Sorbonne Université, ACTION group, Pitié-Salpêtrière Hospital (AP-HP), Paris, France; Laboratoire D’Imagerie Biomédicale (LIB), Sorbonne Université, INSERM, CNRS, Paris, France; Sorbonne Université, Institute of Cardiometabolism and Nutrition (ICAN), Paris, France; Unité D’Imagerie Cardiovasculaire et Thoracique (ICT), Pitié-Salpêtrière Hospital, Paris, France; Laboratoire D’Imagerie Biomédicale (LIB), Sorbonne Université, INSERM, CNRS, Paris, France; Sorbonne Université, Institute of Cardiometabolism and Nutrition (ICAN), Paris, France; European Hospital Georges Pompidou, Assistance Publique – Hôpitaux de Paris (AP-HP)/PARCC, Université Paris-Cité, INSERM, Paris, France; Sorbonne Université, ACTION group, Pitié-Salpêtrière Hospital (AP-HP), Paris, France; Laboratoire D’Imagerie Biomédicale (LIB), Sorbonne Université, INSERM, CNRS, Paris, France; Laboratoire D’Imagerie Biomédicale (LIB), Sorbonne Université, INSERM, CNRS, Paris, France

**Keywords:** deep learning, longitudinal strain, left atrium, feature tracking, CMR

## Abstract

**Aims:**

Feature tracking (FT) is increasingly used on dynamic cardiac magnetic resonance (CMR) images for myocardial strain evaluation but often requires manual initialization, which is tedious and source of variability, especially on the challenging long-axis (LAX) images. Accordingly, we designed a pipeline combining deep learning (DL) with FT for left ventricular (LV) and left atrial (LA) longitudinal myocardial strain estimation.

**Methods and results:**

We studied a multivendor database of 684 individuals divided into: training = 845, tuning = 281, and testing = 116 LAX-CMR cine 2- and/or 4-chamber views. Images were centre cropped. Then, a 2D- and 3D-ResUnet, which considers time as the third dimension, were designed for LV/LA segmentation and used to (i) estimate LV and LA strains (Full 2D−/3D-DL) and (ii) initialize an FT algorithm and further derive LV and LA strains (FT-initialized by 2D−/3D-DL). Left ventricular and LA contours and strain peaks were compared against reference standard (RS) measures performed by an expert using a semiautomated software. Intraclass-correlation-coefficient (ICC) was used to study reproducibility. 3D-DL outperformed 2D-DL segmentation (Dice-scores: 0.94 ± 0.02 vs. 0.90 ± 0.09, *P* = 0.002) and was stable across vendors, field strengths and imaging views. The added value of combining DL with FT was revealed by higher correlations and lower Bland–Altman biases against RS for FT initialized by 3D-DL strains (r ≥ 0.91, |mean-bias|≤0.65%) than for full 3D-DL strains (r ≤ 0.80, |mean-bias|<3.07%). Semiautomated human vs. FT initialized by 3D-DL (ICC ≥ 0.76) and inter-human strain reproducibility was equivalent.

**Conclusion:**

Generalizable DL-based LV and LA segmentation on LAX-CMR images was proposed. Its combination with FT resulted in fully automated and reliable LV and LA strain measures, reaching human reproducibility.

## Introduction

Cardiovascular diseases (CVDs) encompass various disorders, including coronary artery disease, heart failure, stroke, and atrial fibrillation, among others.^1,2^ Cardiovascular diseases early detection is crucial to reduce their subsequent high mortality rates. Imaging techniques including cardiac magnetic resonance (CMR) allow visualization of the heart and quantitative evaluation of cardiac chamber anatomy and function, playing a key role in CVDs diagnosis.

Cardiac magnetic resonance is the reference technique for the evaluation of heart volumes and myocardial mass using the widely available and standardized cine steady-state free precession (SSFP) sequence.^3^ Measurement of left (LV) and right (RV) ventricular volumes using CMR requires a robust and reliable segmentation of heart chambers from a stack of 8 to 12 short-axis (SAX) slices, at end-diastole and end-systole.^4,5^ In addition to SAX orientation, standard CMR exams further include acquisition of long-axis (LAX) images, which are used for the evaluation of left atrial (LA) volumes and function, providing a left heart comprehensive evaluation. Recent studies have highlighted the clinical significance of feature tracking (FT)-derived LV and LA longitudinal strain indices,^6,7^ which can be calculated from LAX cine-CMR images. However, such additional valuable indices are not included in daily clinical routine yet because of the lack of extensive evaluation in large populations due to the need for manual initialization of LV and LA contours on individual images, which is time-consuming and source of variability.

Several research projects including international challenges^8,9^ have been led to automatize heart chamber segmentation from cine CMR images. However, such achievements were mainly focused on SAX images and the clinically valuable LV and RV volumes. As such, automated processing of SAX CMR images improved substantially while using both conventional image processing^10^ and deep learning (DL)-based^11^ algorithms. Progresses on SAX images do not directly apply to LAX images, due to the challenging LV and LA geometry in LAX views compared with the circular LV shape in SAX views, as well as to complex neighbouring structures such as valves and pulmonary veins.

Accordingly, we designed and compared three automated pipelines to segment LV and LA and to estimate LV and LA longitudinal myocardial strains from LAX CMR images: (i) a 2D DL network, (ii) a 3D DL network, including time as the third dimension, (iii) a method combining initialization on a single time phase by the 2D or 3D DL networks with FT.

## Methods

### Population and acquisitions

We retrospectively studied 684 individuals (70.7% men, 55 ± 12 years) including 94 healthy volunteers and 590 patients with various cardiac diseases (myocardial infarction, mitral regurgitation, aortic valve stenosis, familial hypercholesterolaemia), who underwent a CMR exam. These data originate from different protocols approved by local ethics committee (NCT03715998; NCT02517944; healthy volunteers were included from NCT02938910 and NCT02537041)^12–17^ and individual signed informed consent was obtained. Cardiac magnetic resonance data were acquired on either a 1.5T or a 3T CMR scanner from three different vendors. While data from vendors 1 (Siemens healthiness, Erlangen, Germany) and 2 (GE healthcare, Chicago, USA) included both healthy volunteers and patients, vendor 3 (Philips Healthcare, Eindhoven, the Netherlands) data included only patients with myocardial infarction. All CMR exams comprised cine SSFP images in both SAX (stack of 8 to 12 slices) and LAX (2− and 4-chamber single slices) views, which were acquired during consecutive breath-holds using ECG gating and the following average scan parameters: pixel size = 1.29 ± 0.34 mm², acquisition matrix = 180–512 × 200–512, time frames = 25–60, repetition time = 2.76–4.58 ms, echo time = 0.94–2.05 ms, flip angle = 35–77°, slice thickness = 7–8 mm. Of note, LAX images were acquired before contrast injection. Cardiac magnetic resonance data were fully deidentified while restricting DICOM information to image characteristics, scanner vendor, and magnetic field strength.

### Reference standard measures

Experienced operators used commercial software (QMass, Medis Medical Imaging, Leiden, the Netherlands) to estimate (i) LV end-diastolic and end-systolic volumes as well as myocardial mass and LV ejection fraction, derived from delineation of LV endocardial and epicardial borders on the stack of SAX images, while including papillary muscles and trabeculae within the LV blood pool and (ii) LA end-systolic and end-diastolic volumes as well as ejection fraction using the bi-plane area length method, through the delineation of LA borders on both 2− and 4-chamber LAX views, while excluding pulmonary veins and LA appendage.

Long-axis images were further analysed, by an expert blinded to DL developments, using a semiautomated software (CardioTrack, Sorbonne University) dedicated to myocardial strain evaluation based on FT, which was extensively validated on both human^6,18,19^ and small animal^20^ CMR data. Throughout such analysis, contour initialization on a single time phase and subsequent tracking on all remaining phases of the cardiac cycle, as well as strain peak detection were supervised by an expert, and manually corrected if needed. Such semi-automated process and tracking algorithm have been previously described.^19^ The resulting expert measures were used as reference standard (RS) annotations, they comprised LV and LA contours throughout the cardiac cycle on both 2- and 4-chamber LAX views, as well as peak LV global longitudinal strain (LVGLS) and LA global longitudinal strains at the reservoir (LARS), conduit (LACS) and booster (LABS) phases. To account for anatomical continuity (blood pool) and contiguity (LV myocardial wall and blood pool cavity) of left heart structures, the 3 RS annotated structures (LA blood pool, LV myocardium and blood pool) were grouped together in 2 labels defined as ‘blood pool’, which comprised both LV and LA cavities, and ‘LV’ which comprised LV myocardium and cavity.

### Data partition

Data partition into training, tuning, and testing datasets was performed in a pseudo-random manner, ensuring that each dataset was representative of the diversity of LV and LA morphology and function. Indeed, individuals were classified based on LV and LA reference volumes, and ejection fractions. Equivalence among CMR vendors and magnetic field strengths was also considered. Of note, we ensured that images of a given patient were all included either in the training, tuning, or testing dataset. *Table 1* summarizes patient characteristics for the three subgroups (845, 281, and 116 LAX cine views in the training, tuning, and testing sets, respectively), highlighting equal distribution in terms of sex, age, field strengths, vendors, and normal hearts across training and testing datasets. Indeed, LV mass as well as LV and LA volumes and ejection fractions were on average similar between training and testing sets, while high standard deviations highlighted wide left heart size range across all sets. To further evaluate the robustness of our model for under-represented vendors, we conducted a performance test by excluding data from Vendor 3 during model training, while keeping the testing set untouched. Finally, one might note that the number of 2− and 4-chamber views is provided after the exclusion of 126 views, for which expert annotations were missing for either LV or LA due to the presence of artefacts.

**Table 1:** Description of the training, tuning, and testing datasets

	Training	Tuning	Testing	*P* value
Subjects (*n*)	482	142	60	
Views (*n*: 2C/4C)	(845: 425/420)	(281: 140/141)	(116: 56/60)	
Males, *n* (%)	335 (70%)	107 (75%)	42 (70%)	0.501
Age (years)	56 ± 13	56 ± 13	57 ± 13	0.854
Healthy, *n* (%)	58 (12%)	28 (20%)	8 (13%)	0.007
1.5T, *n* (%)	456 (95%)	132 (93%)	54 (90%)	0.331
3T, *n* (%)	26 (5%)	10 (7%)	6 (10%)	
V1, *n* (%)	149 (31%)	54 (38%)	18 (30%)	0.275
V2, *n* (%)	319 (66%)	84 (59%)	38 (63%)	
V3, *n* (%)	14 (3%)	4 (3%)	4 (7%)	
LVM (g)	144.4 ± 51.4	152.3 ± 56.7	134.8 ± 57.0	0.124
LVEDV (mL)	150.0 ± 46.0	155.6 ± 42.9	147.9 ± 50.8	0.457
LVESV (mL)	65.9 ± 30.0	68.2 ± 29.4	64.3 ± 30.8	0.684
LVEF (%)	56.6 ± 11.9	57.3 ± 10.1	55.4 ± 14.8	0.605
LAV max (mL)	71.9 ± 29.1	73.9 ± 27.1	73.4 ± 30.9	0.816
LAV min (mL)	33.1 ± 19.7	33.6 ± 18.2	34.5 ± 20.4	0.891
LAEF (%)	55.8 ± 11.3	55.6 ± 11.5	54.9 ± 12.2	0.888

*P* values are provided for comparisons across training, tuning, and testing sets. Percentages were calculated within each subset (training, tuning, and testing). 2C/4C, 2−/4-chamber views; LAEF, left atrial ejection fraction; LAV max/min, left atrial maximal/minimal volumes; LVEDV/LVESV, left ventricular end-diastolic/end-systolic volumes; LVEF, left ventricular ejection fraction; LVM, left ventricular mass; V1, Vendor 1; V2, Vendor 2; V3, Vendor 3.

### DL segmentation

**Figure 1:**
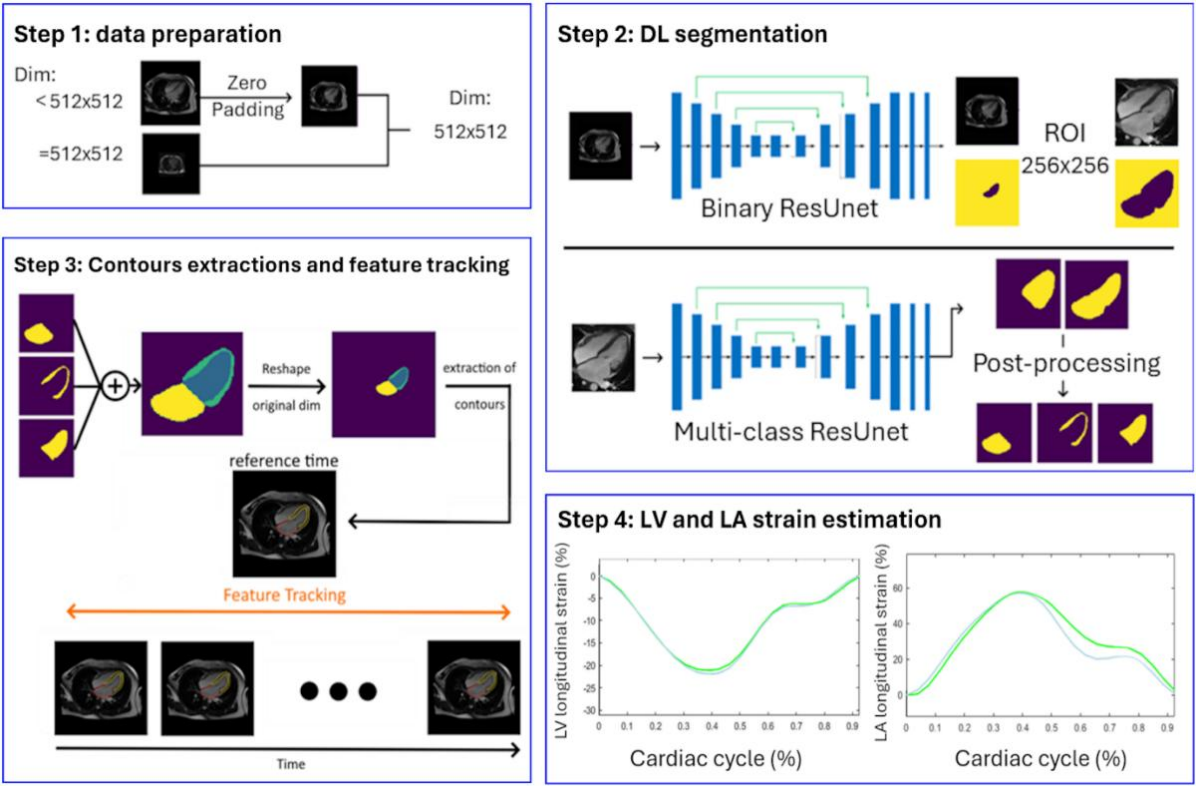
Analysis pipeline for the estimation of LV and LA strain from CMR LAX images. Step 1: data preparation included resizing of all images to 512 × 512 pixels, when needed. Step 2: DL segmentation included dual network used to centre and crop images and then to segment left heart structures. Step 3: resulting DL contours were used on a single reference time to initialize FT, which was then achieved through all remaining phases of the cardiac cycle. Step 4: LV and LA strain waveforms were derived.

CMR data were first prepared by resizing all images to 512 × 512 pixels when needed (*Figure 1*, step 1).

**Figure 2:**
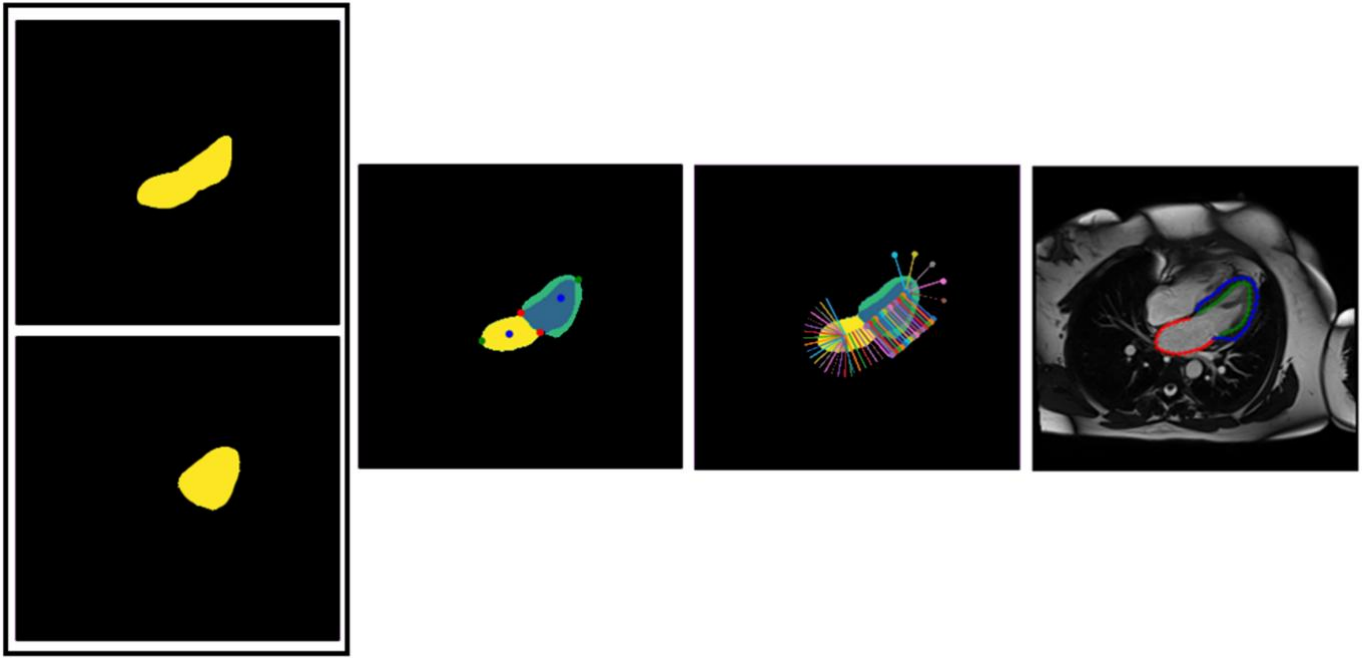
Contour extraction from LV and LA masks. From left to right: predicted labels; landmark definition, landmarks correspond to mitral anulus extremities, LV apex and LA roof, as well as to the point located at 2/3 of the LV long axis length from base to apex and to the LA center of mass; radial detection and equidistant distribution of contour points, as the transition between the heart label (one) and the background (zero); superimposition of contour points on the native CMR image.

A Dual-ResUnet encompassing 2 cascaded U-Net networks^21,22^ was proposed for left heart chamber segmentation on LAX cine CMR images (*Figure 1*, step 2). The ResNet encoder^23^ provided training of a very deep neural network while avoiding issues caused by vanishing or exploding gradients. The first network had a 2D ResUnet architecture and performed a binary segmentation of the heart, which was then used to centre and crop the image around structures of interest, resulting in 256 × 256 images. Such cropping helped focus on key regions to target feature extraction and reduce imbalance between heart and background labels. The second network was designed with either a 2D or a 3D ResUnet architecture and generated a multi-class prediction of the blood pool and LV structures within the cropped image. The 3D architecture was used through time to account for the continuous variation of cardiac structure morphology along the cardiac cycle. For both networks, a Dice binary cross-entropy cost function^24^ was used to manage the overlap between the 2 blood pool and LV labels. These 2 predicted labels were then converted back into LV and LA contours (LA endocardium: LA, LV endocardium: LV Endo and LV epicardium: LV Epi) and were superimposed on the original images (*Figure 2*). To achieve such conversion into individual chamber contours, mitral annulus extremities were first identified as the intersection between predicted LV myocardium and blood pool masks. Then, four other landmarks were defined, including: LV apex and LA roof defined as points of the LV and LA masks located farthest from the mitral annulus centre; the points located within the LV and LA masks, at 2/3 of the LV long axis length from base to apex, as commonly used in American Heart Association LV segment definition,^25^ and at the LA center of mass, respectively. Finally, contour points were extracted by identifying the pixel coordinates where the label intensity transitions from one (predicted mask) to zero (background). This process was performed radially (outwards from the LV and LA chamber central axis) and the identified contours points were resampled equidistantly between landmarks to achieve uniform coverage of LV and LA borders.

### Segmentation evaluation

To evaluate LV Epi, LV Endo, and LA segmentation performance, conventional Dice similarity coefficient and Hausdorff distance were estimated for all cardiac time frames. Time-averaged metrics were then provided for individual datasets. This evaluation was performed for both 2D and 3D ResUnet-derived segmentations. Such segmentations were further compared within subgroups according to vendor, field strength, and acquisition view (2 or 4 chambers).

### DL contours for strain estimation

Two-dimensional or 3D DL-derived LV and LA contours throughout all phases of the cardiac cycle were used to estimate time-resolved strain waveforms using the Lagrangian method, which consists in calculating temporal changes in myocardial longitudinal length, relative to and normalized by early systolic initial length (*Figure 1*, step 4). While LV endocardial and epicardial strain curves were averaged to capture the whole LV myocardial deformation, only the endocardial strain curve was considered for the LA. Finally, DL-derived strain curves were used to extract the previously described LV and LA strain peaks: LVGLS, LARS, LACS, LABS. This processing pipeline was named ‘Full 2D/3D DL’.

### FT initialized by DL for strain estimation

Left ventricular and LA contours at maximal LV and LA dilation were used to initialize previously described^18,19^ FT process (*Figure 1*, step 3). The obtained FT-derived LV and LA contours throughout the cardiac cycle were then used to estimate time-varying longitudinal strain waveforms (*Figure 1*, step 4). Similar to DL-derived strain, such waveforms were used to extract the abovementioned LV and LA strain peaks. This processing pipeline was named ‘FT initialized by 2D/3D DL’.

### Semi-automated human vs machine measure reproducibility

A dataset of 27 unseen patients (54 cine SSFP 2− and 4-chamber views) was used to evaluate model and operator variability. In such evaluation, in addition to the DL analysis, three independent observers including one experienced operator (Operator 1, more than 3 years of expertise) and two operators with intermediate expertise (Operator 2/Operator 3, few months of experience) have used the semi-automated FT software to evaluate LV and LA strain. Of note, this is an internal database including complex cases, in terms of image quality, used for software development and testing.

### Statistical analyses

Continuous variables were provided as mean ± standard deviation, while categorical variables were provided as percentage. Spearman correlation coefficient (r), Student’s *t*-test, and Bland–Altman mean bias and limits of agreement were used to compare LV and LA strain indices obtained using the different DL-based pipelines against expert RS indices. Student’s *t*-test was further used for comparison of segmentation performances between field strengths and imaging views. Three-group comparisons were performed using ANOVA for continuous variables and Pearson chi-square for categorical variables. A *P* value <0.05 was considered as statistically significant. Results related to the 2D network segmentation, along with its direct comparison against the 3D network in terms of strain peaks are provided in the Supplementary material.

Deep learning-based pipeline variability was compared with inter-operator variability through intraclass correlation coefficients (ICC). We used two-way mixed-effects ICC to estimate correlations between individual measurements achieved on the same target patients by fixed human and DL raters.^26^ ICC values below 0.50, between 0.50 and 0.75, between 0.75 and 0.90, and above 0.90 were considered as poor, moderate, good, and excellent, respectively.^27^ Statistical analyses were performed with *JMP* v15.2.0 (SAS Institute Inc., Cary, USA).

### Model description

The first network was trained for 50 epochs with a batch size of 64 images, with an Adam optimizer and a training rate of 0.0001, while evaluating through a Dice binary cross-entropy with logit loss score on the tuning set during training. The 2D/3D multiclass-segmentation networks were respectively trained for 100/100 epochs with a batch size of 64 images/5 cine series, with Adam optimizer and a training rate of 0.001/0.001, while evaluating through a Dice binary cross-entropy with logit loss score on the tuning set during training. All networks were initialized via a truncated normal distribution centred on 0 (He normal initializer) using maxpooling and sigmoid activation, were developed in Python using Pytorch with CUDA 12 and were trained on a computer with the following characteristics: Intel(R) Xeon(R) Gold 6226R CPU @ 2.90GH (cores), 500 GB RAM, 2 NVIDIA RTX A6000 GPU with 48 GB GDDR6 ECC of dedicated memory.

Processing time was recorded for both DL-based and semi-automated analyses.

## Results

A substantial gain in time was observed when using DL-based strategies since processing time was on average less than one minute per patient, while it was lying between 5 and 10 min for semi-automated analysis, depending on operator level of experience, image quality and patient data complexity.

### Left heart chamber segmentation performances

The first binary network contributed to a significant reduction in class imbalance as shown in Supplementary material (Supplementary data online, *Figure S1*). Results of comparison between 3D network multiclass segmentation and expert reference contours are summarized in *Table 2*, revealing satisfactory and equivalent averaged performances for LV endocardial and epicardial walls, and slightly lower performances for the more complex LA endocardial wall. Overall, 97% testing dataset had a mean Dice score ≥ 0.85. The stability of our network was evaluated while re-training three times with random weights, resulting in an overall stable mean Dice score of 0.94. The 2D network failed in depicting either the LA or the LV in 22 views (8 2-chamber and 14 4-chamber views) of the testing set, resulting in a failure rate of 19%, compared with a failure rate of 0% for the 3D network. 2D network overall performances on the remaining views (94 out of the 116 views in testing set) are summarized in the Supplementary material (Supplementary data online, *Table S1*). When compared on the same 94 datasets, the 3D network outperformed the 2D network in terms of mean Dice score (0.94 ± 0.02 vs 0.90 ± 0.09, *P* = 0.002). Supplementary data online, *Video S1* provides an example where the 2D network failed while the 3D network succeeded in left heart segmentation.

**Table 2:** Performances of left heart chamber segmentation for the 3D network

	LV Epi	LV Endo	LA	Mean
Dice score	0.96 ± 0.02	0.94 ± 0.03	0.91 ± 0.10	0.94 ± 0.04
Hausdorff distance (mm)	5.55 ± 2.27	5.57 ± 2.39	6.41 ± 4.0	5.71 ± 2.0

LA, left atrium; LV Endo, left ventricular endocardium; LV Epi, left ventricular epicardium,

Interestingly, multiclass segmentation robustness across CMR field strengths, vendors, and views revealed a slight performance drop in datasets of Vendor 3 as compared with vendors 1 and 2 (*Table 3*). This might be due to lower representativeness since vendor #3 data represented only 3% of the training set (*Table 1*). Finally, our network resulted in equivalent performances through field strengths (1.5 vs. 3T) and imaging views (2− vs. 4-chamber). Overall testing performances when data from Vendor 3 were not seen during training resulted in averaged Dice score that was similar to initial training on all vendors (from 0.94 ± 0.04 to 0.93 ± 0.05).

**Table 3:** Robustness of the 3D network segmentation to differences in magnetic field strength, vendor, and imaging view, in terms of dice score coefficients

	LV Epi	LV Endo	LA	Mean
1.5T	0.96 ± 0.02	0.94 ± 0.03	0.91 ± 0.04	0.93 ± 0.02
3T	0.96 ± 0.01	0.95 ± 0.02	0.90 ± 0.04	0.94 ± 0.02
*P* value	*0.181*	*0.044*	*0.867*	*0.687*
V1	0.96 ± 0.01	0.95 ± 0.02	0.92 ± 0.04	0.94 ± 0.02
V2	0.96 ± 0.01	0.94 ± 0.02	0.91 ± 0.05	0.94 ± 0.02
V3	0.95 ± 0.02	0.92 ± 0.04	0.90 ± 0.03	0.93 ± 0.02
*P* value	*0.072*	*0.034*	*0.561*	*0.056*
2C	0.96 ± 0.01	0.95 ± 0.03	0.90 ± 0.13	0.94 ± 0.05
4C	0.96 ± 0.02	0.94 ± 0.03	0.92 ± 0.03	0.94 ± 0.02
*P* value	*0.672*	*0.372*	*0.131*	*0.301*

*P* values for comparisons between field strengths, vendors and imaging views for all left heart structures are provided. 2C/4C, 2−/4− chamber views; LA, left atrium; LV Endo, left ventricular endocardium; LV Epi, left ventricular epicardium; V1, vendor 1; V2, vendor 2; V3, vendor 3.

### Myocardial strain measurements

**Figure 3:**
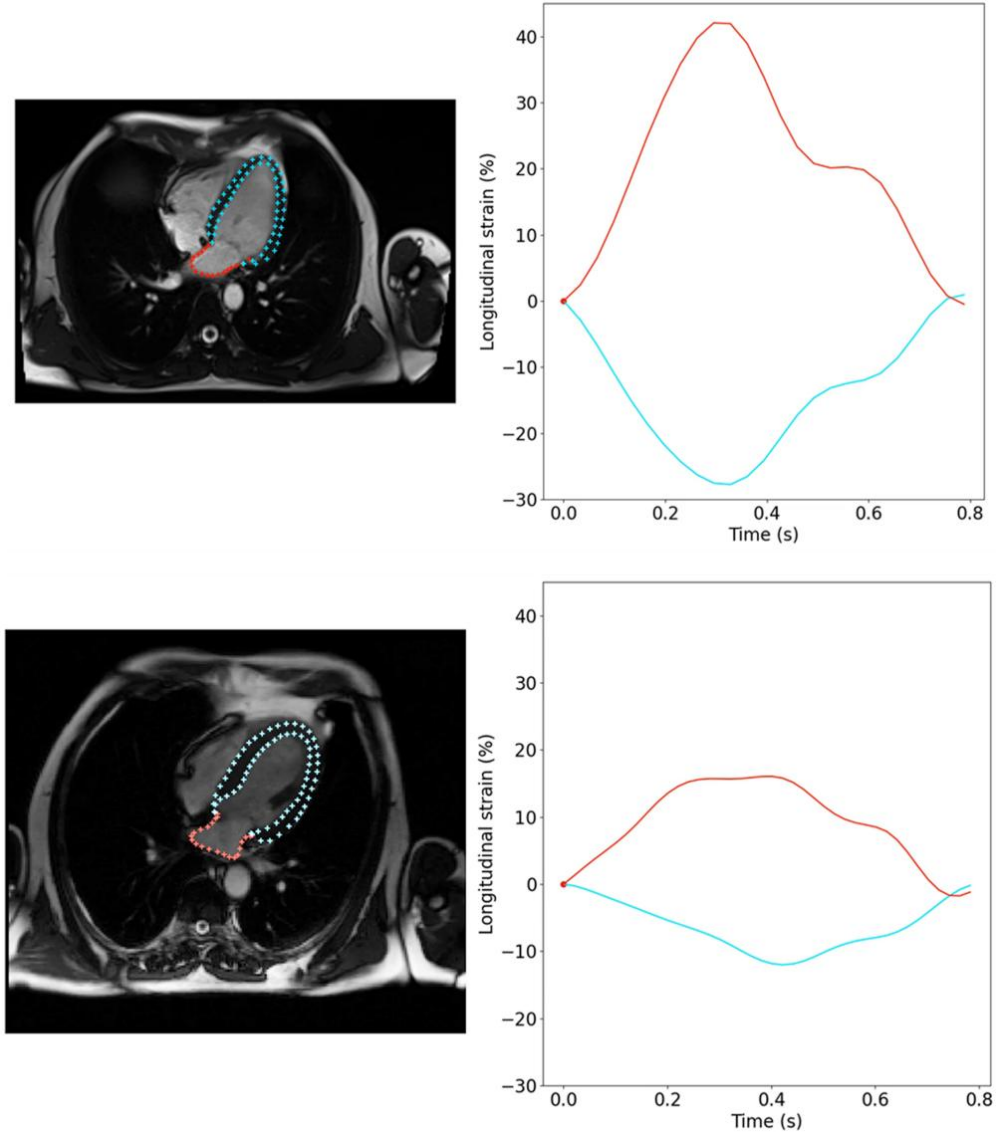
Examples of left heart chamber DL-based contours and corresponding LV and LA time-resolved strain waveforms for a healthy individual, and a patient with abnormal cardiac chambers.

Examples of LV and LA segmentations along with derived strain waveforms were provided for a healthy volunteer and a patient with abnormal heart in *Figure 3* and in Supplementary data online, *Videos S2 and S3*. Left ventricular and LA myocardial strain peaks were provided in supplementary data online, *Table S2* for full 3D DL and in *Table 4* for FT initialized by 3D DL strategies along with correlations as well as Bland–Altman biases and limits of agreement, against RS measures. Such comparisons revealed the added value of combining DL for contour initialization and FT for time-resolved tracking of cardiac chambers, in terms of correlations with RS measures (r ≥ 0.91 for FT initialized by 3D DL and r ≤ 0.80 for full 3D DL) and Bland–Altman biases and limits of agreement, which were narrower for FT initialized by 3D DL strategy compared with full 3D DL. Such added value is even more pronounced for LA strain as compared with LV strain measures. The added value of combining DL with FT was also observed when considering 2D strategies (see Supplementary data online, *Table S2*). Indeed, overall increased correlation coefficients and lower Bland–Altman mean biases were observed for FT initialized by 2D DL as compared with the full 2D DL strategy.

**Table 4:** LV and LA myocardial strain values obtained using FT initialized by 3D DL strategy, along with reference standard expert values

	Reference standard	Prediction	r	*P*-values	µ (LoA)
FT initialized by 3D DL					
LVGLS (%)	−15.0 ± 4.6	−15.7 ± 5.2	0.95	0.82	0.65 (−2.52;3.82)
LARS (%)	28.0 ± 11.1	28.1 ± 11.4	0.94	0.96	−0.08 (−7.9;7.74)
LACS (%)	13.6 ± 7.4	13.3 ± 7.4	0.91	0.81	0.25 (−5.95;6.45)
LABS (%)	13.9 ± 7.1	14.2 ± 7.2	0.92	0.78	−0.29 (−6.04;5.47)

Correlation coefficients, mean Bland–Altman biases, and limits of agreement for comparisons against reference standard were also provided. DL, deep learning; FT, feature tracking; LABS, left atrial global longitudinal strain at the booster phase; LACS, left atrial global longitudinal strain at the conduit phase; LARS, left atrial global longitudinal strain at the reservoir phase; LoA, limits of agreement; LVGLS, left ventricular peak global longitudinal strain; r, Spearman correlation coefficient; µ, mean bias.

### Semiautomated vs. machine measure reproducibility

The 27 individuals included in the reproducibility study comprised both patients and healthy volunteers, resulting in lower but still reasonable overall Dice score (0.87 ± 0.09) obtained using the 3D network as compared with performances on the testing dataset. Semiautomated human analysis vs. FT initialized by 3D DL variability results are summarized in *Table 5* while human vs. full 3D DL comparison results are summarized in Supplementary data online, *Table S3*, revealing good agreement between human operators and FT initialized by DL strategy-derived LV and LA strains. Indeed, such agreement is in the same range as agreement between human operators. In line with strain findings, the full 3D DL strategy resulted in overall moderate agreement with human operators.

**Table 5:** ICC values and confidence intervals for agreement between human operators as well as between human operators and FT initialized by 3D DL in terms of LV and LA myocardial strain values

	Humans	Humans vs. FT initialized by 3D DL
LVGLS	0.92 (0.87;0.95)	0.88 (0.82;0.93)
LARS	0.82 (0.72;0.89)	0.82 (0.73;0.88)
LACS	0.76 (0.64;0.85)	0.80 (0.71;0.87)
LABS	0.75 (0.63;0.84)	0.76 (0.65;0.84)

DL, deep learning; FT, feature tracking; LABS, left atrial global longitudinal strain at the booster phase; LACS, left atrial global longitudinal strain at the conduit phase; LARS, left atrial global longitudinal strain at the reservoir phase; LVGLS, left ventricular peak global longitudinal strain.

## Discussion

A pipeline combining deep learning and feature tracking for automated left ventricular and left atrial longitudinal strain quantification from long-axis cine CMR data was proposed and validated on a large database including data from different CMR vendors, magnetic field strengths, and disease conditions, providing the following main findings: (i) high Dice score coefficients for LV and LA segmentation throughout the cardiac cycle, (ii) high correlations and low Bland–Altman biases for comparisons with reference strain measures, (iii) consistent performances across CMR vendors, field strengths and imaging views, (iv) reproducibility in the same range as semi-automated human measure repeatability, (v) a substantial reduction in processing time. Furthermore, several processing options including 2D and 3D networks, whether combined with FT or not, were tested and all comparative results were provided to highlight the most relevant processing strategy, namely 3D network combined with FT.

Only few CMR studies focused on LAX images^28,29^ and even fewer included both LV and LA segmentation and strain estimation. A previous study^29^ used DL-based segmentation to initialize an FT algorithm and to estimate strain from LAX images on a small group of patients, but only LV strain was reported. Cardiac magnetic resonance studies dedicated to either LV or LA DL-based segmentation on LAX images revealed Dice scores that are in line with ours: Dice scores of 0.93^30^ and 0.929^31^ were achieved for LV segmentation on relatively small and single centre databases, and Dice scores of 0.93,^32^ 0.917,^33^ and 0.90^28^ were achieved for LA segmentation in the literature. While networks reported in^28,33^ were validated on small datasets, evaluation in^32^ was performed on a larger dataset of 600 patients, but all CMR data were acquired at a single centre resulting in a quite homogeneous database. To the best of our knowledge, there are no studies focusing on DL-based strain estimation from LAX CMR images, especially when considering LA tri-phasic strain. Alternatively, there are few echocardiographic DL-based studies^34–36^ focusing on longitudinal strain estimation from LAX images. Other echocardiographic studies focused on LV and LA DL-based segmentation on LAX images, revealing lower to comparable Dice scores than those obtained in our study (0.92 for LV and 0.89 for LA^37^ or 0.912 for LV Endo and 0.855 for LV Epi.^38^)

Segmentation performances tended to be lower on the LA as compared with the LV for both 2D and 3D networks. Such difference could be attributed to the much smaller LA size than LV cavity and to the thinner LA wall as well as more complex geometry, through connections with pulmonary veins and LA appendage. Nevertheless, average LA Dice scores through the entire cardiac cycle were still good. In line with segmentation performances, correlations between DL-based and reference strains were slightly lower for LA tri-phasic strain values than for LV peak systolic strain. Overall, progress from 2D to 3D networks resulted in higher segmentation performances and better agreement in strain measures with the reference. Of note, the 2D network resulted in quite satisfactory results on the LV but not on the LA. Interestingly, FT had a substantial added value whether combined with 2D or 3D network, especially in the LA. This might be explained by the physical integration of heart contraction and relaxation effects on chamber borders, which are continuously accounted for through time when using FT. Indeed, FT has been widely validated on both human^18,39^ and small animal CMR data^20^ and initialization through a robust DL-based segmentation has the advantage of providing an explainable and fully automated processing pipeline for the reliable estimation of both LV and LA strain.

### Clinical implications

In addition to the well-established and clinically relevant LV longitudinal strain,^40^ LA strain has emerged as a sensitive marker of LA early dysfunction, often preceding structural changes such as LA enlargement.^41^ Left atrial strain can be relevant for diagnosis and risk-stratification in conditions such as heart failure, atrial fibrillation, and valvular diseases, where impaired LA strain correlates with adverse outcomes.^42,43^ Indeed, in atrial fibrillation, reduced LA strain predicts stroke risk and post-ablation recurrence.^44^ In myocardial infarction, LA complements LV metric prediction of adverse events.^45^ In such a context, a comprehensive and automated solution quantifying LV and LA strain from LAX CMR images, providing consistent performances across vendors and magnetic field strengths appears to be highly valuable.

### Limitations

The main challenge in the development of DL segmentations is the lack of public databases to provide comparisons with other networks. This is particularly true for CMR LAX images, despite its usefulness for LA volume estimation as well as for LV and LA strain quantification. This explains our first limitation, which resides in the lack of comparisons with other models. However, one might note that such models are widely available for SAX images, but not for LAX images. Additionally, our strain pipeline was not validated on 3-chamber views. This is inherent to strain estimation which is currently performed on 2− and 4-chamber views, rendering annotations on 3-chamber views quite scarce. We believe that with the availability of adequate annotations, our network should adapt to such datasets. Finally, although our database is representative of main CMR vendors and field strengths, it lacks data from low field CMR scanners, which are gaining interest in CMR.^46^

## Conclusion

A robust and generalizable DL-based LV and LA segmentation from long-axis CMR images was proposed and validated in a large database covering different CMR vendors, magnetic field strengths, and disease conditions. Its combination with FT resulted in a fully automated and reliable measurement of LV and LA strain, reaching reliability and reproducibility of semi-automated human analysis.

## Supplementary Material

qyaf070_Supplementary_Data

## Data Availability

The datasets used and/or analysed during the current study are available from the corresponding author on reasonable request.

## Lead author biography

Dr. Nadjia Kachenoura is an Inserm research Director. She was awarded her PhD in biomedical imaging from Université Paris-Sud (2007) and pursued a post-doc fellowship at University of Chicago. There she developed a profile in analysis of cardiac echo, CT and MRI. In 2014, she founded the INSERM/CNRS cardiovascular imaging (LIB-ICV) team at Sorbonne University and became the head of a multidisciplinary group. She has contributed to >200 publications, primarily on the analysis of morphology and haemodynamics of the aorta and the heart and how these relate in a range of cardiovascular diseases. These are widely cited with a H-index of 28. She is PI of several national projects and 2 international projects (USA, Argentina) and a WP leader of several European projects (H2020 MAESTRIA project, EIT Health CMRAI project, SESAME Cohort Project), for a total of €3M funding. She received more than 30 invited talks at international meetings. Since 2014, she hired three permanent positions and supervised 12 PhD students, 3 post-docs (including a H2020 Marie Curie), 6 research engineers and >30 masters students, who gained several awards, highlighting the attractiveness of her group. She also is a member of the European Society of Cardiovascular Radiology and organizing the annual scientific meeting in Paris. She was a member of the e-cardiology working group of the European Society of Cardiology and was member of the National Committee of CNRS in charge of hiring future researchers in the field of health technologies in France (2016-2021). Dr. Kachenoura is committed to international collaborations with the University of Chicago, Johns Hopkins University, University of Favaloro and University of Bologna and is a guest editor in the Journal of Clinical Medicine for a special issue on aortic imaging and a review editor in Frontiers in Medicine Journal.
