# Supplementary material for: Deep learning-based segmentation and strain analysis of left heart chambers from long-axis CMR images
Source: Eur Heart J Imaging Methods Pract. 2025 Jun 3;2(4):qyaf070. doi: 10.1093/ehjimp/qyaf070 (PMC12256142; doi:10.1093/ehjimp/qyaf070)
Supplement: qyaf070_Supplementary_Data [file qyaf070_supplementary_data.zip › Suplementary_final.docx]

**Figure S1.** Whole heart class distribution from native (blue) and center, cropped (orange) images.

**
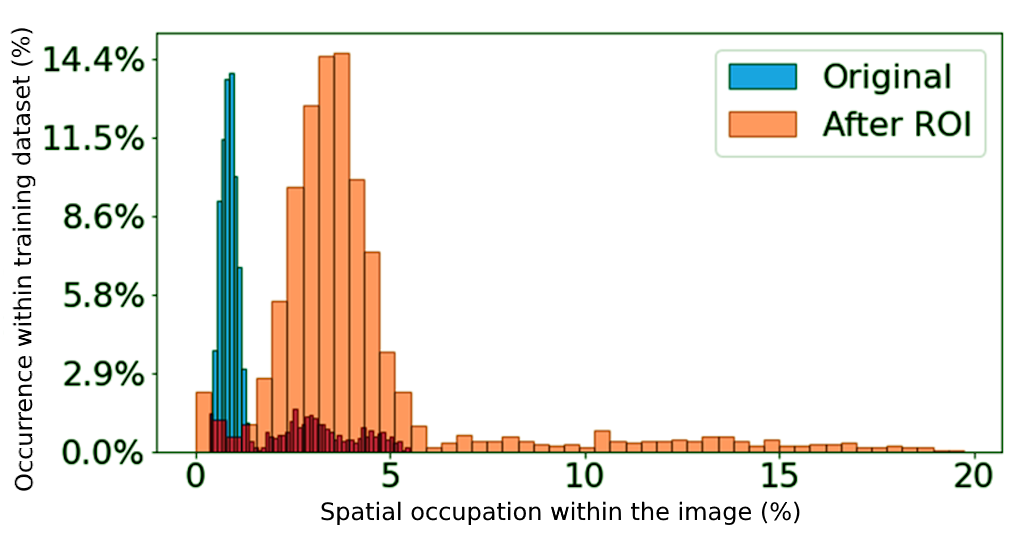
**

ROI: region of interest

**Table S1.** Left heart chamber segmentation performances for the 2D network.

| N = 94 | LV Epi | LV Endo | LA | Mean |
| --- | --- | --- | --- | --- |
| Dice score | 0.93 ± 0.07 | 0.89 ± 0.11 | 0.89 ± 0.10 | 0.90 ± 0.09 |
| Hausdorff distance (mm) | 6.94 ± 5.99 | 7.24 ± 5.53 | 7.66 ± 4.62 | 7.28 ± 5.42 |
| LV Epi: left ventricular epicardium, LV Endo: left ventricular endocardium, LA: left atrium | | | | |

**Table S2.** LV and LA myocardial strain values obtained using the full 2D DL, the FT initialized by 2D DL and the full 3D DL strategies, along with ground truth expert values. Correlation coefficients, mean Bland-Altman biases and limits of agreement for comparisons against ground truth were also provided. Of note, strain results for 2D network were presented on the same 94 out of the 116 views in the testing set as in table S1. The full 3D DL results were presented on the same dataset as the FT initialized by 3D DL.

|  | Ground truth | Prediction | r | p-values | µ (LoA) |
| --- | --- | --- | --- | --- | --- |
| Full 2D DL | | | | | |
| LVGLS (%) | -15.0 ± 4.6 | -15.6 ± 5.5 | 0.79 | 0.37 | 0.68 (-5.92;7.27) |
| LARS (%) | 28.3 ± 10.7 | 39.8 ± 37.6 | 0.32 | 0.01 | -11.53 (-81.36;58.31) |
| LACS (%) | 13.7 ± 6.9 | 18.9 ± 28.6 | 0.28 | 0.10 | -5.16 (-58.96;48.63) |
| LABS (%) | 14.1 ± 7.2 | 20.4 ± 37.3 | 0.26 | 0.11 | -6.31 (-77.08;64.46) |
| FT initialized by 2D DL | | | | | |
| LVGLS (%) | -15.0 ± 4.6 | -15.8 ± 5.2 | 0.95 | 0.23 | 0.87 (-2.26;4.00) |
| LARS (%) | 28.3 ± 10.7 | 29.9 ± 10.9 | 0.84 | 0.30 | -1.67 (-13.67;10.34) |
| LACS (%) | 13.7 ± 6.9 | 14.0 ± 6.9 | 0.75 | 0.73 | -0.35 (-9.99;9.30) |
| LABS (%) | 14.1 ± 7.2 | 15.3 ± 8 | 0.80 | 0.27 | -1.23 (-10.87;8.41) |
| Full 3D DL | | | | | |
| LVGLS (%) | -15.0 ± 4.6 | -15.5 ± 5.0 | 0.80 | 0.48 | 0.48 (-5.5;6.47) |
| LARS (%) | 28.0 ± 11.1 | 30.1 ± 25.2 | 0.54 | 0.44 | -2.15 (-44.02;39.72) |
| LACS (%) | 13.6 ± 7.4 | 13.1 ± 7.9 | 0.52 | 0.67 | 0.46 (-14.24;15.17) |
| LABS (%) | 13.9 ± 7.1 | 17.0 ± 28.6 | 0.32 | 0.31 | -3.07 (-56.39;50.25) |
| DL: deep learning, LVGLS: left ventricular peak global longitudinal strain, LARS: left atrial global longitudinal strain at the reservoir phase, LACS: left atrial global longitudinal strain at the conduit phase, LABS: left atrial global longitudinal strain at the booster phase, FT: feature tracking, r: Spearman correlation coefficient, µ: mean bias, LoA: Limits of Agreement | | | | | |
|  | | | | | |

**Table S3.** ICC values and confidence intervals for agreement between human operators as well as between human operators and full 3D DL, in terms of LV and LA myocardial strain values.

|  | Humans | Humans *vs.* full 3D DL |
| --- | --- | --- |
| LVGLS | 0.92 (0.87;0.95) | 0.70 (0.58;0.80) |
| LARS | 0.82 (0.72;0.89) | 0.65 (0.53;0.77) |
| LACS | 0.76 (0.64;0.85) | 0.44 (0.28;0.59) |
| LABS | 0.75 (0.63;0.84) | 0.52 (0.38;0.67) |
| LVGLS: left ventricular peak global longitudinal strain, LARS: left atrial global longitudinal strain at the reservoir phase, LACS: left atrial global longitudinal strain at the conduit phase, LABS: left atrial global longitudinal strain at the booster phase, DL: deep learning, FT: feature tracking. | | |

**Video file 1.** An example of failed 2D DL segmentation, while the 3D DL segmentation was successful. Left: native images, center: 2D DL masks, right: 3D DL masks. yellow mask: LA, blue and green masks: LV.

**Video file 2.** Example for contours detection and strain measures in a healthy volunteer with normal sized LV and LA. FT initialized by 3D DL contours superimposed on native images (left) along with LV and LA longitudinal strain curves (right). Contours and strain curves are color-coded similarly for LA (red) and LV (blue).

**Video file 3.** Example for contours detection and strain measures in a patient with remodeled LV. FT initialized by 3D DL contours superimposed on native images (left) along with LV and LA longitudinal strain curves (right). Contours and strain curves are color-coded similarly for LA (red) and LV (blue).
